# A nomogram model based on tumor necrosis factor-like ligand 1A(TL1A) and death receptor-3(DR3) promoter methylation for predicting 90-day prognosis in patients with HBV-associated acute-on-chronic liver failure

**DOI:** 10.3389/fmolb.2025.1614311

**Published:** 2025-07-03

**Authors:** Xue-Fei Wei, Feng Zhang, Han-Xu Zhu, Zhe-Zhe Tian, Miao-Miao Xu, Yu-Chen Fan, Kai Wang

**Affiliations:** ^1^ Department of Hepatology, Qilu Hospital of Shandong University, Jinan, China; ^2^ Department of Hepatology, Qilu Hospital of Shandong University (Qingdao), Qingdao, China

**Keywords:** HBV-ACLF, methylation, noninvasive model, prognosis, TL1A/DR3

## Abstract

**Purpose:**

Acute-on-chronic liver failure (ACLF) associated with hepatitis-B-virus (HBV) is a life-threatening condition characterized by severe hepatic dysfunction. The TL1A/DR3 signaling axis modulates immune responses and contributes to hepatic inflammation. This study aimed to investigate the methylation level of TL1A/DR3 promoter, explore its ability to predict prognosis, and establish a prognostic model combined with clinical indicators.

**Method:**

Methylation status and gene expression of TL1A and DR3 were analyzed in peripheral blood mononuclear cells (PBMCs) from 714 participants using Methylight and quantitative polymerase chain reaction (qPCR). Univariate, LASSO, and multivariate analyses were performed to identify key prognostic factors for 90-day outcomes in patients with HBV-associated acute-on-chronic liver failure (HBV-ACLF) and develop corresponding prognostic models. Model performance, including calibration and clinical utility, was evaluated using receiver operating characteristic (ROC) curves, Hosmer-Lemeshow (H-L) tests, and decision curve analysis (DCA). A visual nomogram was constructed to integrate these factors for risk stratification.

**Result:**

Analysis revealed significantly reduced TL1A and DR3 promoter methylation in HBV-ACLF patients, correlating with impaired liver function and coagulation parameters. PBMCs from these patients showed elevated mRNA expression of TL1A, DR3 and IL-6 compared to other groups. Methylation levels of TL1A and DR3 demonstrated high sensitivity and specificity in predicting HBV-ACLF severity. Besides, non-survivors exhibited lower TL1A/DR3 methylation than survivors. A prognostic model integrating prothrombin time activity (PTA), procalcitonin (PCT), and TL1A/DR3 methylation demonstrated excellent performance in predicting 90-day outcomes.

**Conclusion:**

Aberrant TL1A/DR3 promoter methylation reflects the disease severity, and can serve as potential biomarkers for the risk assessment of HBV-ACLF.

## Introduction

Chronic liver disease (CLD) is a major public health problem in the world (Keegan et al., 2025). Acute-on-chronic liver failure (ACLF) is a syndrome with acute jaundice deepening and coagulation dysfunction as manifestations of liver failure caused by various inductions on the basis of chronic liver disease. It may be combined with hepatic encephalopathy, ascites, electrolyte disorders, infection and other complications (Sarin et al., 2019; Hartmann et al., 2023). In Asia, chronic hepatitis B virus (HBV) infection is the most important risk factor for cirrhosis and liver failure, accounting for more than 70% (Wong and Chan, 2020). The excessive immune response induced by HBV activity is a major factor driving the progression of chronic hepatitis B (CHB) and/or cirrhosis (LC) to ACLF and exacerbates short- and medium-term mortality in patients (Zhao et al., 2018). Therefore, there is an urgent need for a non-invasive, efficient and reliable biomarker for the early detection and diagnosis of HBV-ACLF and for the establishment of an effective prognostic assessment model for disease surveillance, clinical management and reduction of the short-term mortality in patients with HBV-ACLF.

Immune-mediated inflammatory damage plays an important role in the pathogenesis and prognosis of ACLF, with systemic inflammatory responses often induce organ failure through the “inflammation-injury” cascade effect (Khanam and Kottilil, 2021). Cytokine storm, as characteristic pathological mechanism of HBV-ACLF, stems from the disorder of pro-inflammatory and anti-inflammatory effects caused by the imbalance of immune system homeostasis, releasing a large amount of cytokines such as IL-6, TNF-α, and IL-1β, and triggering systemic injury, multiple organ failure or death. Among them, the cytokine storm induced by viral infection can interfere with the host’s inflammatory regulation mechanism, preventing it from effectively eliminating inflammation (Jarczak and Nierhaus, 2022; Fajgenb et al., 2020). Excessive inflammatory responses driven by immune imbalance enhance susceptibility to viruses, thereby exacerbating the risk of organ dysfunction and death in patients with ACLF (Casulleras et al., 2020). Tumor necrosis factor-like cytokine 1A (TL1A), a member of the tumor necrosis factor (TNF) superfamily protein, is encoded by TNFSF15 and plays a key role in immune regulation, recruitment of inflammatory cells, and promotion of T cell activation and differentiation (Mig et al., 2002; Meng et al., 2023). As one of the functional receptors of TL1A, DR3, also known as the death receptor 3, is an important member of the TNFR protein superfamily and is mainly expressed in activated antigen-presenting cells, lymphocytes, and fibroblast-like cells (Papadakis et al., 2004; Bamias et al., 2013). After TL1A/DR3 binding, it can pathway synergistically promotes T cell activation, activates NF-κB pathway, induces apoptosis inhibitory proteins, thereby promoting the transcription and release of inflammatory factors TNF-α and IL-6, and participating in the regulation of immune response and the development of inflammatory diseases, include rheumatoid arthritis (RA), asthma, and autoimmune diseases (Aiba et al., 2014; Schmitt et al., 2024). When the NF-κB pathway is continuously overactivated, it leads to the “cascade amplification” release of various pro-inflammatory factors, forming a cytokine storm and exacerbating systemic inflammatory damage (Yamamoto and Gaynor, 2001). Recent studies have found that TL1A/DR3 plays an important role in many liver diseases such as non-alcoholic fatty liver disease (NAFLD) and liver fibrosis (Guo et al., 2019; Luo et al., 2021). TL1A/DR3 signaling can promote the secretion of GM-CSF, increase the infiltration of myeloid cells, and cause tissue inflammatory damage (Li et al., 2019). It was found in animal models of primary biliary cirrhosis (PBC) and related liver fibrosis that the expression level of TL1A was associated with the severity of inflammation (Guo et al., 2019; Luo et al., 2021; European Association for the Study of the Liver, 2018). However, its role in expression and prognosis in HBV-ACLF remains unclear.

Recent studies have shown that DNA methylation in peripheral blood mononuclear cells (PBMCs) holds potential as a complementary biomarker for multiple diseases (Li et al., 2025). In addition, we found significant abnormalities in PBMCs DNA methylation profiles in patients with chronic hepatitis and alcoholic liver disease and NAFLD (Zachou et al., 2022). It is considered an ideal biomarker for disease detection and prognostic prediction. Therefore, we designed an experiment to detect the methylation status of TL1A and DR3 promoters in PBMCs of HBV-ACLF patients, HBV-LC patients, CHB patients and HCs. We further analyzed the differences in methylation expression between the 3-month death group and the survivor group. Finally, we comprehensively evaluate the potential clinical value of TL1A and DR3 promoter methylation levels as non-invasive biomarkers for the diagnosis and short-term prognosis of HBV-ACLF. The purpose of this series of studies is to provide a more reliable scientific basis for the prediction and prognosis assessment of HBV-ACLF.

## Materials and methods

### Study population

**FIGURE 1:**
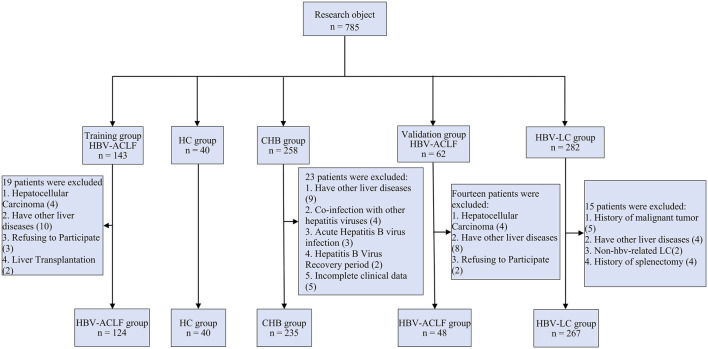
Flowchart of research object selection.

This study collected study samples from Qilu Hospital of Shandong University, Qilu Hospital of Shandong University (Qingdao adopt), and Shandong Public Health Clinical Research Center. The samples included 172 patients with HBV-ACLF 266 HBV-related LC, 253 patients with CHB, and 40 healthy controls (HCs), subject selection as shown in Figure 1. Diagnostic criteria for patients with HBV-ACLF according to the Asia Pacific Association for the Study of the Liver (APASL) Guidelines (updated 2019) (Sarin et al., 2019). LC stands for early compensated liver cirrhosis and is defined based on liver stiffness measurements and laboratory indicators. CHB patients are defined as chronic necrotic-inflammatory disease of the liver caused by persistent HBV infection, HBsAg positive for at least 6 months (European Association for the Study of the Liver, 2018). HCs who tested negative for viral hepatitis and had no evidence of other liver or malignant disease served as normal controls. Demographic, clinical, and laboratory data were recorded after enrollment. The symptoms and signs of the patients were closely monitored, and regular examinations and follow-up visits were conducted according to the individual clinical conditions of the patients. The end point of observation was 90 days. Informed consent of all patients was obtained in advance and approved by the Medical Ethics Committee of Qilu Hospital of Shandong University (Ethics review number: 2021-S923).

### Extraction of PBMCs, RNA and DNA

Peripheral blood was collected from all participants on the first day after diagnosis. PBMCs were separated by adding lymphocyte separation solution with density gradient centrifugation method. PBMCs at the middle interface were collected and washed twice with PBS. Total RNA and DNA was extracted from PBMCs using 1 mL TRIzol Reagent (Invitrogen). Eluted RNA was extracted with PrimerScript™RT Reagent Kit (Perfect Real Time; Legumes, Japan), according to the instructions to reverse transcribe RNA into cDNA. The extracted DNA was eluted with 50–100 μL of sodium hydroxide.

### DNA modification by sulfate and MethyLight

DNA bisulfate modification was performed using EZ DNA Methylation gold kit (Zymoresearch, Orange, CA, USA) according to the instructions, and modified DNA was obtained after elution. Two sets of sulfites transformed DNA primers and probes were designed and generated by Shanghai Sangon Co., Ltd. to quantitatively detect MethyLight. The specific primers and probe sequences are shown in Table 1. The system was then prepared with EpiTect MethyLight PCR + ROX vial kit (including positive and negative controls) and methylation-specific PCR was performed. β-actin gene as a normalized reference set. In addition, in order to ensure the repeatability of the experiment, we set up three biological replicates, and each sample was repeated three times. The results of MethyLight were calculated based on percentage methylation reference value (PMR).

$$\begin{array}{ll} & PMR=100\%\times 2exp.\;\left[Delta\;Ct\;\left(target\;gene-control\;gene\right)\right.\\ & \;\left.\times \;Sample-Delta\;Ct\;\left(target\;gene-control\;gene\right)M.SssI-Reference\right] \end{array}$$

(Su et al., 2024).

**TABLE 1:** Primer sequence.

Gene	Forward primer (5′-3′)	Reverse primer (5′to 3′)	Probe sequence
RT-PCR			
TL1A	GAAATGACAGTATCTGCGGAGTTTA	CAACTAGCTACTGTCTGGCACTGG	
IL-6	ATGCAATAACCACCCCTGAC	GAGGTGCCCATGCTACATTT	
DR3	CAACTCCACCTGCCTTGTGTGT	CCACTGCTGAACAGTTCTCCAG	
β-actin	TGGTGATGGAGGAGGTTTAGTAAGT	AACCAATAAAACCTACTCCTCCCTTAAA	
Methylight			
TL1A	ATTTAGTTAGGACGTATATAGGTG	TTTACCAATTTAACAAACCCGAA	CAACTCCACCTGCCTTGTGTGT
DR3	TATTTTGTGTTTTTGGTCGTAGTAGGTA	CCCTCTACTCGACCTAAACCTAA	ACGACAACGAACAAACCAAATAAAACACGCGAA
β-actin	TGGTGATGGAGGAGGTTTAGTAAGT	AACCAATAAAACCTACTCCTCCCTTAAA	ACCACCACCCAACACACAATAACAAACACA

### Real-time PCR

The mRNA expression levels of TL1A, DR3 and other related inflammatory factors in PBMCs of each group were detected by fluorescence quantitative PCR. The primers sequences are shown in Table 1. Results The method of comparison (2−ΔCt, ΔCt = Ct (target)-Ct (β-actin)) was used.

### Predictor selection and prediction model development and validation

To identify the predictors in the prognostic model, i.e., the risk factors associated with the short-term prognosis of HBV-ACLF. We used the “glmnet” software package (Lei et al., 2023) to screen the optimal predictors from the clinical parameters of the training cohort through univariate logistic regression analysis and LASSO regression 10x cross-validation analysis. To verify and evaluate the clinical accuracy and applicability of the model, we evaluated the receiver operating characteristic curve (ROC). Hosmer-Lemeshow (H-L) goodness of fit test and decision curve analysis (DCA) was used to evaluate the calibration and clinical practicability of the model. Finally, Nomogram to show variable relationships.

### Statistical analysis

SPSS 29.0, R (4.1) for data analysis and GraphPad Prism 10 for visualization. Numerical variables are expressed in medians and quartiles. Categorical variables are represented by numbers (%). Continuous variables were compared by Student t test, Mann-Whitney U test and Kruskal-Wallis H test. Spearman rank correlation test was used to analyze the correlation between TL1A and DR3 methylation and clinically relevant indicators. Chi-square test was used to compare binary categorical variables between the two groups. The difference was statistically significant with P < 0.05 (bilateral).

## Result

### Study the general characteristics of the population

In our study, a total of 714 participants were included after subject screening. For 172 patients with HBV-ACLF, we used randomization as a simple and straightforward internal cross-validation method, assigned to the training (n = 124) or validation (n = 48) cohort in a ratio of 7:3. General clinical data of HCs, CHB, HBV-LC and HBV-ACLF patients were collected as shown in Table 2, including gender, age, alanine aminotransferase (ALT), aspartate aminotransferase (AST), total bilirubin (TBIL), albumin (ALB), prothrombin time (PT), platelet (PLT), International normalized ratio (INR), prothrombin time activity (PTA), creatinine (Cr), and alpha-fetoprotein (AFP), Log10 [HBV-DNA], ammonia, hepatic encephalopathy (HE), white blood cell (WBC) count, procalcitonin (PCT), ascites, bacterial infection, smoking history, alcohol consumption history, MELDs, PMR, and 3-month mortality.

**TABLE 2:** General clinical characteristics of study subjects.

Variable	HCs (N = 40)	CHB (N = 253)	HBV-LC (N = 267)	HBV-ACLF training (N = 124)	HBV-ACLF validation (N = 48)
Age	33.5 (27.5, 42.5)	43 (35, 51)	53 (46, 61)	50.00 (40.00, 59.75)	49.00 (43.00, 56.00)
Gender, man (%)	23 (57.5%)	200 (79.05%)	186 (69.66%)	84 (67.7%)	37 (77.1%)
ALT	10.00 (7.00, 12.25)	32 (18, 76)	31 (22, 61)	91.50 (36.25, 241.50)	95.00 (42.50, 341.75)
AST	8 (6, 10)	25 (21, 45)	42 (29, 77)	96.00 (59.50, 182.50)	110.00 (53.50, 249.50)
TBIL	6.85 (4.70, 10.20)	12.30 (9.10, 15.90)	32.40 (15.90, 62.20)	235.55 (156.75, 318.75)	238.55 (140.65, 401.00)
ALB	44.80 (43.53, 46.80)	48.20 (45.50, 49.80)	34.30 (30.20, 40.70)	32.70 (29.85, 35.68)	31.95 (29.57, 35.38)
PLT	219.50 (201.75, 238.25)	201 (170, 241)	66 (46, 121)	101.50 (63.00, 139.00)	81.00 (50.00, 154.75)
PT	11.95 (11.725, 12.175)	12.65 (12, 13.125)	15.95 (14.98, 17.1)	20.55 (18.60, 27.03)	24.10 (19.62, 28.48)
INR	0.93 (0.92, 0.97)	0.98 (0.96, 1.07)	1.27 (1.12, 1.50)	1.79 (1.58, 2.31)	2.04 (1.71, 2.41)
PTA	90 (86, 94)	102 (90, 106)	69 (54, 86)	41.00 (31.00, 48.00)	36.50 (28.25, 45.00)
Cr	43 (40, 51)	48 (43.5, 54.5)	51.05 (44.75, 54.9)	55.00 (41.50, 65.00)	60.00 (45.00, 69.75)
AFP	NA	3.21 (2.38, 4.60)	8.07 (3.56, 32.82)	28.60 (6.59, 136.38)	14.03 (4.43, 69.94)
log10 [HBVDNA]	NA	2.3 (1.3, 4)	3.7 (2.14, 4.76)	4.18 (3.24, 5.32)	3.77 (3.31, 5.82)
Ammonia	NA	NA	NA	56.00 (46.00, 81.00)	59.50 (48.00, 92.00)
WBC	6.25 (5.32, 7.15)	6 (4, 7.75)	6.3 (4.2, 9.8)	7.62 (5.30, 11.04)	7.46 (5.31, 10.15)
PCT	NA	NA	0.2 (0.155, 0.285)	0.50 (0.30, 0.97)	0.47 (0.28, 1.05)
MELDs	NA	NA	14 (10.32, 17.68)	18.09 (15.56, 22.86)	20.42 (16.35, 23.93)
PMR TL1A	7.45 (6.18, 9.34%)	5.53 (4.16, 7.41)	3.48 (2.86, 4.27)	1.86 (1.04, 2.94)	1.62 (0.99, 2.77)
PMR DR3	74.44 (65.93, 81.98)	63.17 (57.06, 69.55)	57.32 (52.29, 63.57)	56.87 (48.24, 63.74)	55.24 (48.76, 60.48)
90-day mortality, n (%)	NA	NA	NA	54 (43.5%)	27 (56.2%)
Ascites, n (%)	0	0	177 (66.29%)	77 (62.1%)	33 (68.8%)
HE, n (%)	0	0	31 (11.61%)	26 (21.0%)	12 (25.0%)
Infection, n (%)	NA	NA	NA	70 (56.5%)	27 (56.2%)
Smoking history, n (%)	18 (45%)	122 (48.22%)	116 (43.45%)	42 (33.9%)	23 (47.9%)
Drinking history, n (%)	22 (55%)	146 (57.71%)	151 (56.55%)	53 (42.7%)	28 (58.3%)

### The expression differences of TL1A and DR3 promoter methylation in PBMCs of HBV-ACLF, CHB, HBV-LC and HCs groups

**FIGURE 2:**
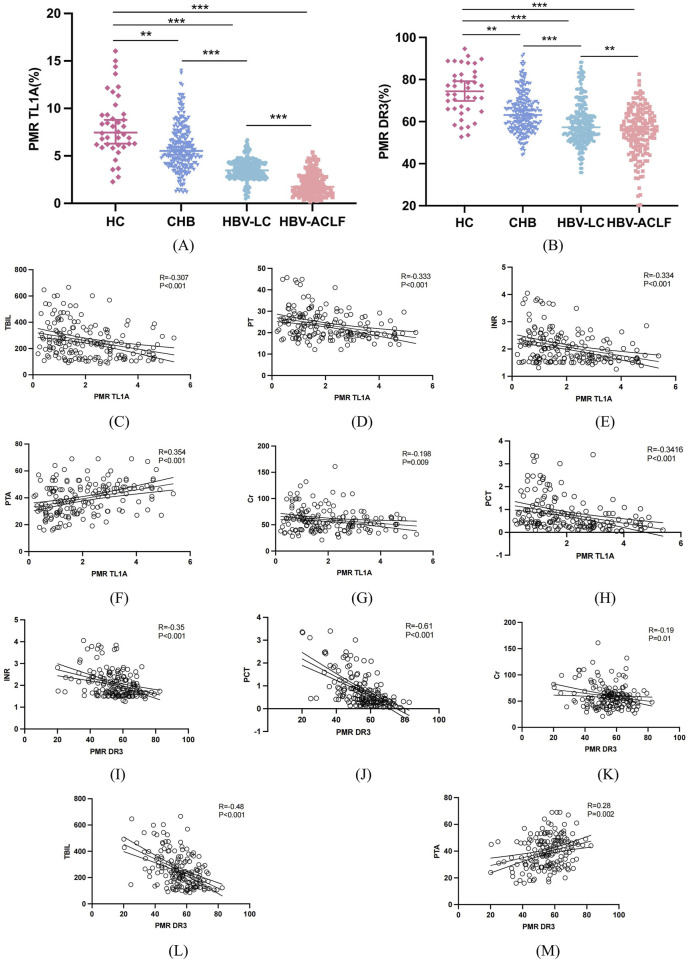
Expression difference of TL1A and DR3 promoter methylation in HBV-ACLF, HBV-LC, CHB, HCs and correlation analysis with clinical indicators. **(A,B)** Expression of TL1A and DR3 promoter methylation in PBMCs of HBV-ACLF, HBV-LC, CHB and HCs groups. **(C–H)** Correlation analysis between TL1A methylation level and TBIL, PTA, PT, INR, Cr, PCT. **(I–M)** Correlation analysis between DR3 methylation level and TBIL, PTA, INR, Cr, PCT.

As Figures 2A,B shows, we analyzed TL1A and DR3 promoter methylation levels (PMR values) in PBMCs of HBV-ACLF, HBV-associated LC, CHB, and HCs groups. TL1A methylation in HBV-ACLF patients (median 1.74%, 1.04%–2.92%) was significantly lower than in HBV-LC (median 3.48%, 2.86%–4.27%), CHB (median 5.53%, 4.16%–7.41%, P < 0.001), and HCs (median 7.45%, 6.18%–9.34%, P < 0.001). Also, CHB had lower TL1A methylation than HCs (P = 0.009).

The DR3 promoter methylation are shown that HBV-ACLF patients (median 56.13%, 48.47%–63.26%) was significantly lower than that in patients with HBV-LC (median 57.32%, 52.29%–63.57%, P < 0.001). HBV-LC patients had significantly lower than CHB patients (median 63.17%, 57.06%–69.55%, P < 0.001) and HCs patients (median 74.44%, 65.93%–81.98%, P < 0.001).

### Correlation between the methylation status of TL1A and DR3 promoters in PBMCs of HBV-ACLF patients and clinicopathological features

To determine whether TL1A and DR3 promoter methylation is influenced by such as age, sex, and infection levels. We grouped patients by basic and clinicopathological features (Table 3). Methylation levels were significantly reduced in men, over 50 years, patients with bacterial infections, and those with HE. However, there was no significant correlation between their methylation and ascites. Spearman rank correlation test found that TL1A promoter methylation were significantly negatively correlated with TBIL (R = −0.307, P < 0.001), PT (R = −0.333, P < 0.001), INR (R = −0.334, P < 0.001), PCT (R = −0.3416, P < 0.001), and Cr (R = −0.198, P = 0.009), and positively with PTA (R = 0.354, P < 0.001), with no significant correlation to other indicators. DR3 promoter methylation was negatively related to TBIL (R = −0.48, P < 0.001), INR (R = −0.35, P < 0.001), PCT (R = −0.61, P < 0.001), Cr (R = −0.19, P = 0.01), and positively to PTA (R = 0.28, P = 0.002). The methylation levels of TL1A and DR3 promoters are closely related to key clinical indicators such as liver function, coagulation function, infection and renal function (Figures 2C–M).

**TABLE 3:** Methylation levels of TL1A and DR3 and diagnostic value of MELDs in HBV-ACLF.

Parameter	Sensitivity	Specificity	Jorden index	AUC	95% CI
PMR TL1A	98.83%	95.84%	0.595	0.841	0.787–0.896
PMR DR3	91.86%	86.8%	0.536	0.830	0.762–0.899
MELDs	87.79%	82.06%	0.411	0.742	0.679–0.805

### Differences of mRNA expression levels of TL1A, DR3 and related inflammatory cytokines in PBMCs of different groups

**FIGURE 3:**
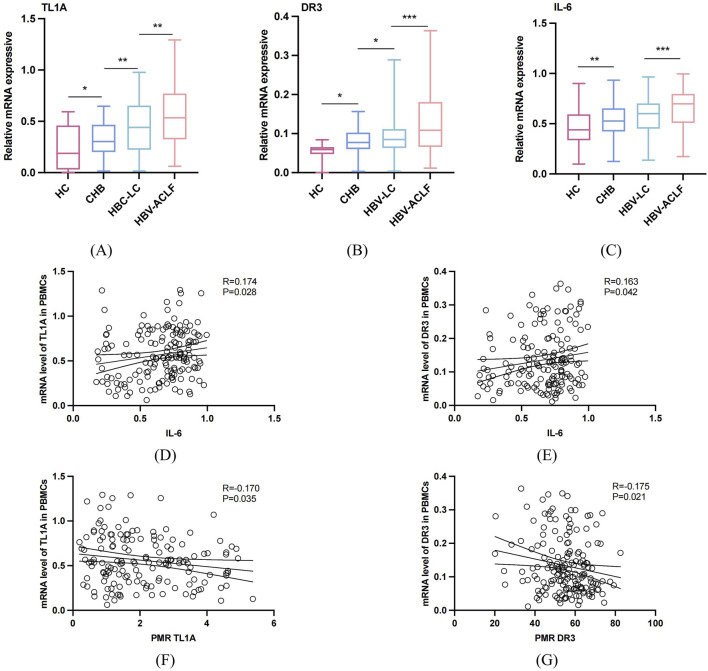
The mRNA expression levels of TL1A, DR3 and related cytokines in PBMCs of different groups, correlation with promoter methylation status and inflammation level. **(A–C)** The mRNA expression levels of TL1A, DR3 and IL-6 in PBMCs in HBV-ACLF, HBV-related LC, CHB and HCs groups were compared. **(D,E)** The mRNA expression correlation analysis of TL1A, DR3 and IL-6. **(F,G)** Analysis of the expression levels of TL1A and DR3 mRNA and their correlation with PMR.

As Figures 3A–C shows, RT-PCR detected mRNA levels of TL1A, DR3, and IL-6 in PBMCs. HBV-ACLF patients (median 0.534, 0.325–0.764); (median 0.109, 0.066–0.178); (median 0.699, 0.510–0.77) had significantly higher expressions than the HBV-LC group (median 0.440, 0.226–0.651, P = 0.0011). (median 0.085, 0.063–0.111, P < 0.001); (median 0.6, 0.450–0.702, P < 0.001), which were higher than the CHB (median 0.302, 0.205–0.456, P = 0.0018); (median 0.526, 0.425–0.651, P < 0.001); (median 0.077, 0.06–0.01, P = 0.028), and the CHB group higher than HCs (median 0.18, 0.03–0.440, P = 0.039); (median 0.440, 0.338–0.591, P = 0.007); (median 0.059, 0.048–0.065, P < 0.001). As shown in Figures 3D–G, correlation analysis revealed a significant negative correlation between TL1A/DR3 mRNA and their promoter methylation. (P = 0.035, 0.021). Spearman analysis found significant positive correlation between TL1A/DR3 mRNA and IL-6, (P = 0.028, 0.042), suggesting TL1A and DR3 are related to inflammation.

### Evaluate the role of TL1A and DR3 promoter methylation in predicting HBV-ACLF

**FIGURE 4:**
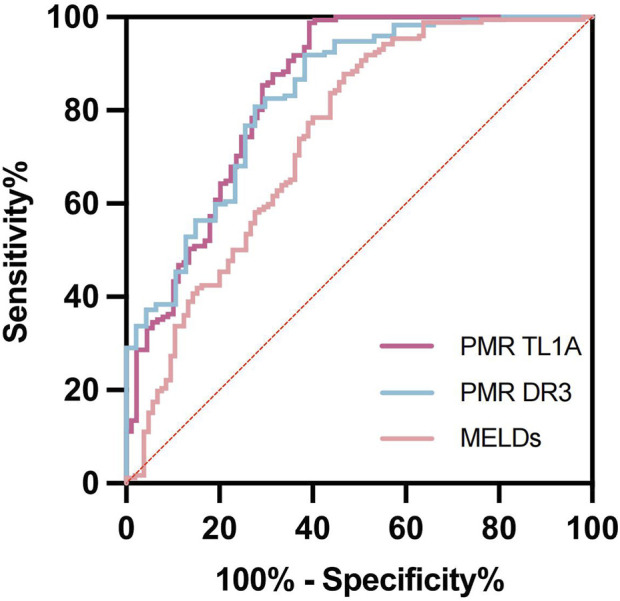
TL1A, DR3 methylation levels and MELDs evaluated the ROC curve of HBV-ACLF.

The ROC curve evaluated the diagnostic value of TL1A and DR3 methylation level, and MELDs, as shown in Figure 4; Table 4, the PMR AUC of TL1A promoter was 0.841, and DR3 promoter was 0.830, higher than MELD score (AUC 0.742). The optimal cut-off points of TL1A and DR3 methylation levels were 4.901% and 69.44%, respectively, which showed high sensitivity (98.83%, 91.86%) and specificity (95.84%, 86.80%) in predicting disease status, and could effectively distinguish patient groups.

**TABLE 4:** Relationship between clinicopathological features and methylation levels of TL1A and DR3 promotors.

Clinicopathological features	N	PMR (TL1A)	PMR (DR3)	P value
Gender				
Man	121	1.622 (0.998, 2.767)	55.066 (46.655, 62.044)	0.036, 0.016**a
Female	51	2.181 (1.353, 3.319)	59.367 (53.80, 64.447)	
Age				
≤50	86	1.561 (0.981, 2.777)	54.007 (45.219, 61.431)	0.039, 0.032*a
>50	86	1.904 (1.182, 3.416)	57.284 (51.665, 65.152)	
AFP				
>20	89	1.70 (1.022, 2.822)	56.004 (48.146, 63.559)	0.916, 0.93b
≤20	83	1.811 (1.067, 2.941)	56.937 (50.077, 62.928)	
HE				
Positive	38	1.128 (0.864, 1.861)	48.369 (42.232, 55.845)	0.0025, 0.0009**a
Negative	134	2.028 (1.178, 3.031)	57.476 (51.521, 63.921)	
Ascites				
Positive	110	1.795 (1.044, 2.923)	55.487 (48.393, 61.216)	0.692, 0.361b
Negative	62	1.694 (1.049, 2.866)	59.03 (49.684, 65.365)	
Bacterial infection				
Positive	97	1.469 (0.976, 2.618)	53.956 (48.223, 60.337)	0.007, 0.0382*a
Negative	75	2.220 (1.277, 3.466)	59.188 (50.190, 66.171)	

### Differences between TL1A and DR3 promoter methylation in 90-day outcomes in patients with HBV-ACLF

**FIGURE 5:**
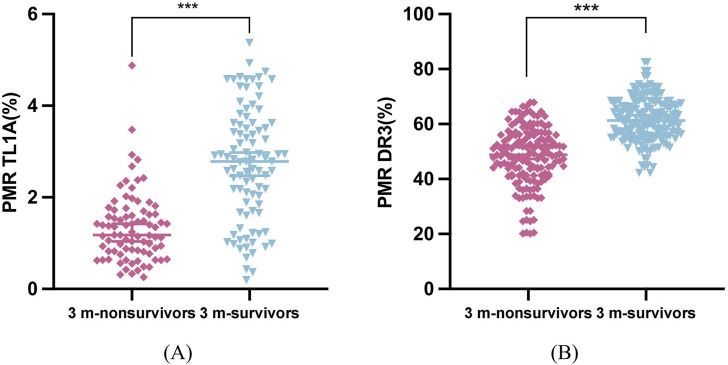
Differences in methylation levels of TL1A and DR3 among 90 days of follow-up. **(A)** TL1A promoter PMR values in survivor and non-survivor patients at 3 months **(B)** DR3 promoter PMR values in survivor and non-survivor patients at 3 months.

After 3 months of follow-up, 81 of 172 HBV-ACLF patients died, with a 90-day mortality rate of 47.09% (81/172). TL1A methylation levels in the death group (median 1.18%, 0.822%–1.63%) were significantly lower than those in the survivor group (median 2.78%, 1.77%–3.60%; P < 0.001). The DR3 methylation level in the death group (median 48.78%, 41.60%–56.0%) was significantly lower than that in the survivor group (median 61.32%, 55.71%–67.07%; P < 0.001) (Figures 5A,B).

### Baseline characteristics and risk factors were selected based on 90-day prognosis of HBV-ACLF patients

**FIGURE 6:**
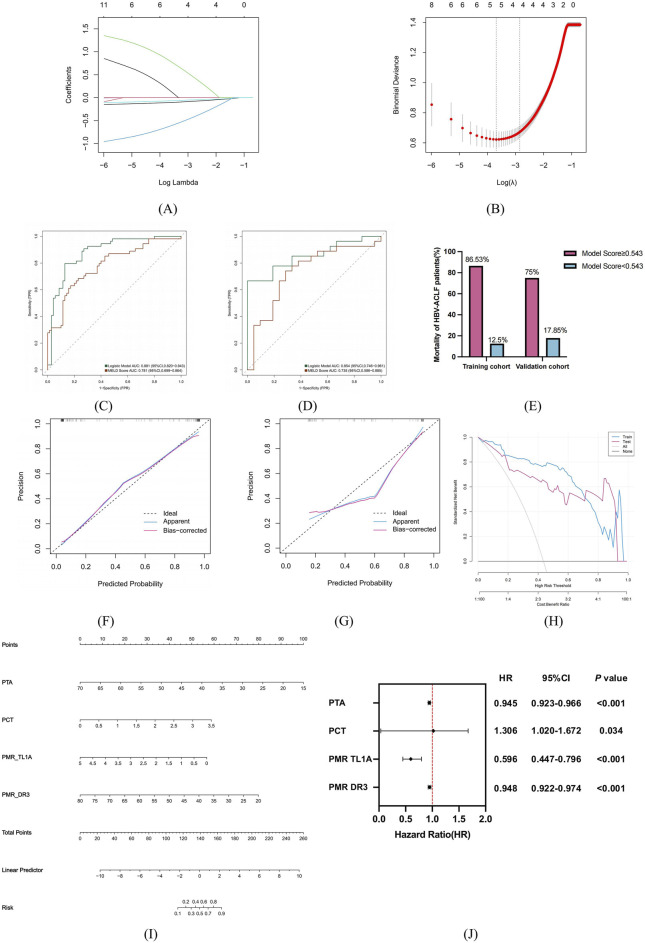
The risk factors were screened according to the 90-day prognosis of HBV-ACLF patients, and the prognosis model was established and evaluated. **(A)** Variation characteristics of variable coefficients. **(B)** The process of selecting the optimal value of parameter λ in Lasso regression model by cross-validation method. **(C,D)** Trained and validated ROC characteristic curves of predictive models for HBV-ACLF patients in the cohort. **(E)** Mortality of HBV-ACLF patients in the training and validation groups distinguished by model score cutoff values. **(F–G**) H-L calibration curves for training and validating predictors in the cohort. The X-axis represents the probability of prediction and the Y-axis represents the probability of observation. The dashed line represents the perfect prediction of the ideal model, and the solid line represents the calibration diagram of the predicted model. **(H)** Predict the DCA of the model in the training and validation queue. The red and blue lines represent the net benefit to patients when the model is used in a clinical intervention. **(I)** Nomogram to predict 90-day prognosis of patients with HBV-ACLF. **(J)** Multivariate Cox proportional hazard regression analyses of prognostic factors associated with 90-day mortality in patients with HBV-ACLF.

HBV-ACLF patients in training (n = 124) and verification (n = 48) groups were divided into survivor and non-survivor groups per 90-day prognosis (Table 5). In the training group, non-survivor group had higher TBIL, PT, INR, WBC, PCT, bacterial infection, HE, MELDs and lower PLT, TL1A, DR3 promoter methylation, PTA (P < 0.05). In the verification group, non-survivor group’s TBIL, ammonia, Cr, PCT, TL1A, DR3 promoter methylation levels, MELDs results were like the training group (P < 0.05), suggesting these may be risk factors for poor prognosis. Univariate logistic regression on training cohort clinical features found 12 possible predictors (Table 6). These 12 factors went through LASSO regression with 10-fold cross-validation (Figures 6A,B), and PTA, PCT, PMR (TL1A) and PMR (DR3) were selected as optimal variables at 1se = 0.056. Multivariate logistic regression analysis was performed for these four variables, and the P-values were all less than 0.05.

**TABLE 5:** Baseline characteristics of HBV-ACLF patients based on 90-day prognosis.

Variable	HBV-ACLF training			HBV-ACLF validation		
	Survival	Un-survival	p	Survival	Un-survival	p
Age	49.50 (40.00, 56.50)	51.00 (43.25, 61.25)	0.287	48.00 (36.00, 57.50)	50.00 (48.00, 56.00)	0.142
Gender, (man) (%)	46 (65.7%)	38 (70.4%)	0.582	16 (76.2%)	21 (77.8%)	1.000
ALT	93.00 (31.00, 230.25)	86.00 (37.75, 247.00)	0.689	95.00 (32.50, 396.50)	136.00 (45.00, 278.00)	0.486
AST	96.00 (55.00, 193.00)	92.50 (62.50, 179.25)	0.630	89.00 (59.50, 181.50)	141.00 (47.00, 272.00)	0.603
TBIL	204.25 (129.52, 268.30)	264.10 (202.10, 365.97)	0.001	214.10 (133.50, 262.85)	322.90 (167.00, 474.00)	0.033
ALB	33.20 (29.68, 35.82)	32.10 (30.03, 35.32)	0.681	33.70 (29.30, 39.00)	31.50 (29.50, 33.20)	0.117
PLT	108.00 (67.00, 162.00)	78.50 (49.50, 118.50)	0.009	81.00 (65.50, 183.50)	78.00 (48.00, 103.00)	0.197
PT	19.15 (17.38, 21.30)	26.35 (21.53, 31.30)	<0.001	22.50 (19.50, 26.30)	26.90 (19.70, 31.20)	0.067
INR	1.66 (1.51, 1.88)	2.29 (1.78, 2.74)	<0.001	1.97 (1.68, 2.25)	2.21 (1.71, 2.47)	0.149
PTA	47.00 (41.00, 53.00)	31.50 (26.00, 38.50)	<0.001	38.00 (31.00, 46.50)	31.00 (28.00, 44.40)	0.061
Cr	53.00 (42.50, 62.25)	56.00 (40.75, 79.00)	0.203	54.00 (41.00, 63.50)	65.00 (48.00, 89.00)	0.047
AFP	31.52 (6.04, 129.90)	26.35 (7.03, 149.35)	0.811	14.13 (4.87, 256.40)	9.25 (3.67, 51.24)	0.216
Log10 [HBVDNA]	3.93 (3.18, 4.94)	4.37 (3.24, 5.42)	0.195	3.58 (3.15, 6.53)	4.01 (3.31, 5.82)	0.685
Ammonia	53.50 (45.00, 74.25)	61.00 (48.75, 85.50)	0.132	55.00 (41.50, 69.00)	77.00 (50.00, 99.00)	0.027
WBC	6.47 (4.39, 10.28)	8.76 (6.31, 11.62)	0.014	6.72 (5.30, 9.71)	7.79 (5.64, 12.23)	0.377
PCT	0.36 (0.23, 0.56)	0.90 (0.52, 1.70)	<0.001	0.30 (0.24, 0.51)	0.81 (0.44, 1.89)	<0.001
MELDs	16.52 (14.39, 18.34)	22.38 (18.17, 25.16)	<0.001	17.32 (14.67, 21.00)	22.33 (19.45, 26.84)	0.006
PMR TL1A	2.80 (1.87, 3.62)	1.16 (0.63, 1.58)	<0.001	2.62 (1.40, 3.44)	1.39 (0.89, 1.81)	0.003
PMR DR3	61.88 (56.26, 67.57)	48.33 (41.32, 55.91)	<0.001	58.29 (55.24, 66.67)	51.10 (45.19, 57.00)	<0.001
Ascites, n (%)	42 (60.0%)	35 (64.8%)	0.584	7 (33.3%)	8 (29.6%)	0.784
HE, n (%)	7 (10.0%)	19 (35.2%)	<0.001	3 (14.3%)	9 (33.3%)	0.131
Bacterial infection, n (%)	34 (48.6%)	36 (66.7%)	0.044	12 (57.1%)	15 (55.6%)	0.912
Smoking history, n (%)	25 (35.7%)	17 (31.5%)	0.621	10 (47.6%)	13 (48.1%)	0.971
Drinking history, n (%)	30 (42.9%)	23 (42.6%)	0.976	12 (57.1%)	16 (59.3%)	0.883

**TABLE 6:** Potential prognostic predictors of HBV-ACLF patients were analyzed based on univariate and multivariate logistic regression.

Variable	Univariate		Multivariate	
	OR (95%CI)	p	OR (95%CI)	p
Ascites	1.228 (0.589, 2.562)	0.584		
HE	4.886 (1.871, 12.761)	0.001		
Bacterial infection	2.118 (1.016, 4.415)	0.045		
Smoking history	0.827 (0.389, 1.758)	0.622		
Drinking history	0.989 (0.483, 2.028)	0.976		
Gender (man)	1.239 (0.577, 2.662)	0.583		
Age	1.020 (0.990, 1.050)	0.193		
PTA	0.846 (0.800, 0.896)	0.000	0.855 (0.792, 0.923)	0.000
AST	1.000 (0.999, 1.002)	0.631		
TBIL	1.005 (1.002, 1.009)	0.002		
PMR TL1A	0.236 (0.141, 0.397)	0.000	0.376 (0.175, 0.810)	0.013
PMR DR3	0.838 (0.788, 0.893)	0.000	0.892 (0.810, 0.981)	0.019
PT	1.296 (1.174, 1.432)	0.000		
INR	15.081 (5.133, 44.303)	0.000		
ALT	1.000 (1.000, 1.001)	0.467		
Cr	1.019 (1.000, 1.037)	0.045		
AFP	1.000 (1.000, 1.000)	0.512		
Log10 [HBVDNA]	1.223 (0.957, 1.561)	0.107		
Ammonia	1.009 (0.998, 1.020)	0.123		
WBC	1.058 (0.984, 1.137)	0.130		
PCT	10.133 (3.730, 27.528)	0.000	4.257 (1.132, 16.017)	0.032
MELDs	1.333 (1.193, 1.489)	0.000		
ALB	1.001 (0.927, 1.082)	0.975		
PLT	0.994 (0.988, 1.000)	0.039		

### Establishment and evaluation of short-term prognostic model for HBV-ACLF patients

Finally, according to the results of multivariate logistic regression analysis, PTA, PCT, PMR (TL1A) and PMR (DR3) were used to establish a clinical model to predict the 90-day prognosis of HBV-ACLF patients. The formula is as follows:

$$Logit\left(P\right)=13.042+\left(-0.157\times PTA\right)+1.449\times PCT+\left[-0.978\times PMR\left(TL1A\right)\right]+\left[-0.115\times PMR\left(DR3\right)\right],$$

where P is the patient’s death risk probability.

As shown in Figures 6C,D, the ROC curve was drawn to evaluate the discriminability of the model. In the training cohort, the model had an AUC of 0.881, and the validation group model had an AUC of 0.854, stable around 0.85. When the AUC are both greater than 0.8, and the difference is less than 0.1, it indicates that the overall effect in the model is good, reaching the practical level (Belkin et al., 2019). The Jorden index was 0.668, the sensitivity was 0.796, and the specificity was 0.871. At the optimal cut-off of 0.543, in the training cohort, patients with a model score≥0.543 had an 86.53% mortality rate, and those <0.543 had a 12.5% rate. In the validation cohort, the rates were 75% and 17.85% respectively (Figure 6E). The LR model had better diagnostic efficiency than MELDs (training: AUC 0.781; validation: AUC 0.735). These results indicate logistic regression models can be used for data set classification.

To further clarify whether the model is clinically usable. H-L goodness of fit test and calibration curves showed good fit between prediction and actual probabilities. The results show that in the training group and the verification group, the prediction probability and the actual probability of the model fit well and have high prediction accuracy. DCA results indicated patients could benefit from clinical intervention. The results showed that the threshold was between 0.08 and 0.99 for the training group and 0.21–0.99 for the validation group. In the training and validation queues, LR showed a higher net benefit (Figures 6F–H). Finally, the four prediction factors are synthesized by nomogram to show the relationship between the variables in the prediction model (Figure 6I), and the multivariate Cox proportional hazards analysis showed that the hazard ratio associated with the risk factors: PTA (HR = 0.945, 95%CI:0.923–0.966), PCT (HR = 1.306, 95%CI:1.020–1.672), PMR TL1A (HR = 0.596, 95%CI:0.447–0.796), PMR DR3 (HR = 0.948, 95%CI:0.922–0.974) (Figure 6J).

## Discussion

In this study, we first reported that TL1A and DR3 promoter methylation levels in PBMCs of HBV-ACLF patients were significantly reduce. Meanwhile, their mRNA expression levels were higher and correlated with inflammation. Clinicopathological analysis showed that in HBV-ACLF patients, male gender, age over 50, infection, and HE were associated with reduced TL1A and DR3 methylation. Also, the methylation levels correlated with key clinical indicators like TBIL, PTA, INR, and Cr, suggesting they can help indicate liver disease status. As non-invasive serological markers, ROC curves showed that TL1A and DR3 methylation outperformed MELDs in predicting HBV-ACLF. At the same time, at the 3-month follow-up endpoint, lower TL1A and DR3 methylation levels were seen in the death group. We constructed a short-term prognosis model for HBV-ACLF using PTA, PCT, PMR (TL1A), and PMR (DR3), which was more effective than MELDs in predicting prognosis, as demonstrated by ROC curve, H-L fitting, and DCA.

At present, the pathogenesis of HBV-ACLF is mainly closely related to immune imbalance and cytokine storm. The overly activated immune response in the patient’s body can trigger the cascade release of pro-inflammatory factor cytokines forming typical pathological characteristics of cytokine storm, which in turn leads to liver function damage, organ failure, and significantly increases the risk of death (Fajgenb et al., 2020). Therefore, early prediction is key to reducing cytokine storm and improving survival for HBV-ACLF patients. TL1A, as a key factor regulating the inflammatory network, its overexpression can upregulate the expressions of TNF-α and IL-1β in liver tissues and macrophages (Papadakis et al., 2004). DR3 binding TL1A activates the NF-κB pathway to drive the explosive secretion of pro-inflammatory factors and amplifies the Th17 cell-mediated inflammatory response through the JAK1/STAT3 pathway (Bamias et al., 2025; Perks et al., 2016). DNA methylation as a major epigenetic modification is of great value in the early detection and prognosis of diseases (Tao et al., 2024; Heeke et al., 2024). Studies have confirmed that the methylation of GPX4, SOCS1 and other genes is related to the occurrence and prognosis of HBV-ACLF (Su et al., 2024; Li et al., 2023). Studying the dynamic properties of methylation makes it a viable target for therapeutic intervention (Zhao et al., 2013; Zeybel et al., 2015). Detectable changes in DNA methylation levels occur early in the disease, more significantly than changes in MELDs (Cheishvili et al., 2024). Therefore, in order to explore the expression of TL1A/DR3 in PBMCs of patients with HBV-ACLF and determine its predictive value for the prognosis of HBV-ACLF by detecting the methylation level. In this study, we quantitatively assessed the expression levels of TL1A and DR3 mRNA and promoter methylation in HBV-ACLF patients using blood samples, and concluded that TL1A and DR3 showed high expression and hypomethylation in HBV-ACLF. The reduced promoter methylation leads to increased mRNA expression of TL1A and DR3, which finding may not only be due to the hypomethylation of the CpG island in the promoter region, thereby exposing the transcription factor binding sites. It is also possible to regulate the participation of transcription factor activity mediated by miRNA (Ma et al., 2015; Cedar and Bergman, 2009).

In addition, we compared promoter methylation of the inflammatory-related molecules TL1A and DR3 in the 90-day survivor and death groups of HBV-ACLF patients. We found that the methylation of TL1A and DR3 was lower in the death group, and the ROC curve of the model was significantly higher than the MELDs in predicting the prognosis of HBV-ACLF. These results suggest that TL1A and DR3 may be involved in the occurrence of HBV-ACLF and the pathophysiological mechanisms related to prognosis and the degree of inflammation. It is suggested that the methylation of TL1A and DR3 in PBMCs can be used as biomarkers for evaluating the prognosis of HBV-ACLF, providing a new non-invasive detection method for predicting the prognosis of liver failure. It is also worth noting that TL1A and DR3 promoter methylation was detected only in PBMC and not in liver tissue, mainly because this study was designed to evaluate the diagnostic value of TL1A and DR3 methylation levels as non-invasive biomarkers, which are easily accessible and the extracted DNA is stable for long-term preservation for analysis. In addition, peripheral blood monocytes, part of the immune system’s front line, resulting in the same changes in related genes in PBMC (Mohr and Liew, 2007). The simple and effective new diagnostic method provides an opportunity for early monitoring and treatment of patients with ACLF, thus delaying the progression of the disease.

The proinflammatory state of local liver inflammation and systemic inflammatory response syndrome (SIRS) are critical drivers of mortality in HBV-ACLF patients (Triantafyllou et al., 2018). PCT is key to early recognition of severe infections and sepsis, reflecting the severity of systemic inflammatory responses (Wen et al., 2024). Multiple studies have shown that high serum PCT levels are an independent risk factor for death in patients with HBV-ACLF, which is consistent with our findings (Wang et al., 2025). Therefore, PCT plays an important role in the development of inflammation in patients with HBV-ACLF. At present, there are several reports on HBV-ACLF prognosis prediction biomarkers and models. Yang et al., built a 3-month prognosis model based on five factors: age, TBIL, PTA, lymphocyte percentage and monocyte percentage (Yang et al., 2022). These studies based on laboratory indicators, overlook the acute inflammatory response status of patients. Previous studies found peripheral blood gene methylation in HBV-ACLF related to prognosis, but no applicable clinical model (Li et al., 2023; Wang et al., 2025). Through quantitative detection, analysis and screening, we found that the following predictors were related to prognosis: PCT, PTA, PMR TL1A and PMR DR3, and constructed a short-term prognosis model of HBV-ACLF. The model uses four readily available clinical variables to rapidly predict the prognosis of HBV-ACLF, and fully considers the clinical practical value of convenience, low trauma and high feasibility, showing its potential in clinically assisted decision making and optimized resource allocation. Since this article focuses more on clinical research, mainly seeking biological markers related to the prognosis of patients with HBV-ACLF, future studies will deeply explore the mechanism of TL1A/DR3 promoter methylation in the pathogenesis, progression and prognosis of HBV-ACLF.

It is worth noting that this study has some limitations and deficiencies that need to be improved. Firstly, due to the high risk of liver biopsy in patients with HBV-ACLF, there was a lack of verification of TL1A/DR3 promoter methylation in liver tissues in the study. In future studies, we will validate both HBV-ACLF tissue and hematological assays to confirm our conclusion. Secondly, the sample size of this study still needs to be further expanded, potential confounding factors such as selection bias caused by single-center samples and sensitivity limitations of some detection methods in micro-samples may slightly affect the analysis results. Therefore, multi-center, large-sample and prospective follow-up studies need to be carried out in the future to further confirm the results of this study. Finally, the molecular mechanism by which TL1A/DR3 is involved in the disease progression and prognosis of HBV-ACLF remains unclear and requires further study.

In summary, this study further emphasizes the significant role of TL1A and DR3 methylation levels in important non-invasive biomarkers for predicting the onset and prognosis of HBV-ACLF, and it indicates that this nomogram model has the potential to evaluate the prognosis of HBV-ACLF. It plays an important role in guiding the monitoring and treatment of clinical high-risk groups, thereby reducing the mortality of patients, and providing new ideas and support for its development as a clinical therapeutic target in the future.

## Data Availability

The original contributions presented in the study are included in the article/supplementary material, further inquiries can be directed to the corresponding author.
